# Prevalence of chronic kidney disease in Peruvian primary care setting

**DOI:** 10.1186/s12882-017-0655-x

**Published:** 2017-07-19

**Authors:** Percy Herrera-Añazco, Alvaro Taype-Rondan, María Lazo-Porras, E. Alberto Quintanilla, Victor Manuel Ortiz-Soriano, Adrian V. Hernandez

**Affiliations:** 1 Escuela de Medicina, Universidad Peruana de Ciencias Aplicadas, Olavegoya 1879 dpto 701 Jesus Maria, Lima, Peru; 2 Hospital Nacional Dos de Mayo, Lima, Peru; 3 CRONICAS Centro de Excelencia de Enfermedades Crónicas, Universidad Peruana Cayetano Heredia, Lima, Peru; 4 Centro de Atención Integral de Diabetes e Hipertensión (CEDHI) – EsSalud, Lima, Peru; 5 Facultad de Salud Pública y Administración, Universidad Peruana Cayetano Heredia, Lima, Peru; 6 University of Connecticut/Hartford Hospital Evidence-based Practice Center, Hartford, CT 06102 USA; 7 Unidad de Conocimiento y Evidencia (CONEVID - UPCH), Lima, Peru

## Abstract

**Background:**

Chronic Kidney Disease (CKD) is a worldwide public health problem. There are few studies in Latin America, especially in primary care settings. Our objective was to determine the prevalence, stages, and associated factors of CKD in primary care setting.

**Methods:**

We did a retrospective secondary analysis of a database from the Diabetes and Hypertension Primary Care Center of the Peruvian Social Security System (EsSalud) in Lima, Peru. We defined CKD as the presence of eGFR <60 mL/min and/or albuminuria >30 mg/day in 24 h, according to Kidney Disease: Improving Global Outcomes (KDIGO). Factors associated with CKD were evaluated with Poisson Regression models; these factors included age, gender, type 2 diabetes mellitus (DM2), hypertension (HTN), body mass index (BMI), and uric acid. Associations were described as crude and adjusted prevalence ratios (PR) and their 95% confidence intervals (95% CI).

**Results:**

We evaluated 1211 patients (women [59%], mean age 65.8 years [SD: 12.7]). Prevalence of CKD was 18%. Using the estimated glomerular filtration rate (eGFR), the prevalence was 9.3% (95% CI 5.3 – 13.3) in patients without HTN or DM2; 20.2% (95% CI 17.6 – 22.8) in patients with HTN, and 23.9% (95% CI 19.4 – 28.4) in patients with DM2. The most common stages were 1 and 2 with 41.5% and 48%, respectively. Factors associated with CKD in the adjusted analysis were: age in years (PR = 1.03, 95% CI 1.01 – 1.04), DM2 (PR = 3.37, 95% CI 1.09 – 10.39), HTN plus DM2 (PR = 3.90, 95% CI 1.54 – 9.88), and uric acid from 5 to <7 mg/dL (PR = 2.04, 95% CI 1.31 – 3.19) and ≥7 mg/dL (PR = 5.19, 95% CI 3.32 – 8.11).

**Conclusions:**

Prevalence of CKD in the primary care setting population was high. CKD is more frequent in the early stages of the disease, and individuals with hypertension, DM2, older age and hyperuricemia have higher prevalence of CKD.

**Electronic supplementary material:**

The online version of this article (doi:10.1186/s12882-017-0655-x) contains supplementary material, which is available to authorized users.

## Background

Chronic Kidney Disease (CKD) is a worldwide public health problem with an estimated prevalence of 10 to 13% in high-income countries [1–4]. However, it is estimated that 80% of people with CKD live in low and middle-income countries such as those in Latin America [5].

CKD epidemiologic characteristics in low and middle-income countries have been poorly defined due to the scarce number of population studies, inconsistent diagnostic methods of renal function measurement and variable quality of the studies [6].

Some population-based studies have evaluated CKD prevalence in countries such as México [7], El Salvador [8], Nicaragua [9], and Perú [10]. In the first two countries, authors assessed semi-quantitative proteinuria and estimated Glomerular Filtration Rate (eGFR) calculated by the Modification Diet in Renal Disease (MDRD), while in Nicaragua the GFR was calculated by MDRD. Although study samples were not representative of the general population, prevalence of CKD was 13% in Nicaragua, 18% in El Salvador, 22% in Mexico City and 33% in Jalisco, Mexico [7–9].

In Peru, a population-based study conducted in the provinces of Lima and Tumbes used the CKD-Epidemiology Collaboration (CKD - ​​EPI) formula to calculate the GFR and proteinuria/creatinine ratio. This study found that the prevalence of CKD in the general population was 16.8%; however, CKD stages were not determined [10].

Although some studies have found that about 95% of people with CKD belong to stages 1 to 3 [11], medical care costs increase with disease progression, with considerably higher expenses in patients with stages 4 and 5 [12]. Also, the cost of patients with stage 5 requiring some form of renal replacement therapy (RRT) represent a challenge for low and middle-income countries as health systems of these countries usually do not have financial resources to provide this coverage [13].

Peru is a Latin American country with a fragmented and deficient health system, with a health expense per capita of $283, below the Latin American average, which was $625 in 2011 [14]. Although the Ministry of Health of Peru has improved coverage of patients requiring hemodialysis (HD), this still is insufficient [15]. Therefore, there is a need to estimate the prevalence of CKD and its different stages to develop an accurate projection of resources [12, 13, 15, 16].

The objective of this study was to determine the prevalence, stages and factors associated with CKD in a primary care population, as an effort to contribute to the knowledge of the epidemiology of this disease in low and middle-income countries and to be a reference for the distribution of resources to these patients.

## Methods

### Study design

We performed a retrospective secondary analysis of database from the Diabetes and Hypertension Primary Care Center (Centro de Diabetes e Hipertension [CEDHI]) of the Peruvian Social Security System (EsSalud) in Lima, Peru. The CEDHI is a specialized center of EsSalud, member of the Social Security Health System belonging to the first level of care. This center is attended by patients aged 18 and over, with the diagnosis of Type 2 Diabetes Mellitus (DM2), Hypertension (HTN) or without these diseases, who are referred annually from the primary care centers attached to the Rebagliati - EsSalud care network in Lima. All the evaluation is done in 48 h and the patient returns to his/her care center of origin, with results and treatment. This situation prevents the patient from having to wait an extended time for their consultations or lab exams.

### Study population

We included in the analysis all patients treated at the CEDHI from January 1st to August 31st, 2011, as in this period medical records were recorded electronically. We excluded patients without information to calculate eGFR (i.e. unavailable age, sex or creatinine). Also, those who did not have albuminuria results were excluded as this is required to assign stages according to the Kidney Disease: Improving Global Outcomes (KDIGO). Similarly, we excluded those with urinary tract infection for the possibility of producing a false positive of albuminuria, and pregnant women as they present a physiological increase of albuminuria levels.

### Procedures

During the study period, all patients from the CEDHI were tested for albuminuria (assessed with a 24-h urine collection before their appointment), serum uric acid and creatinine. To calculate the eGFR we used the Modification Diet Renal Disease formula of 4 variables (MDRD-4).

Additionally, weight, height, and blood pressure measurements were performed by a group of four trained nurses. They measured weight and height on a standard scale with height rod (Detecto®, USA), without shoes, in a standing position with light clothing. Blood pressure was measured with a standing mercury sphygmomanometer (nova-presameter® - Riester, Jungingen, Germany) after 15 min of rest, having asked patients not to consume coffee before evaluation.

Serum albumin and all biochemical tests were performed in an automated laboratory from the *Programa de Atencion Domiciliaria – EsSalud*, with the Konelab™ PRIME 60 Clinical Chemistry Analyzer equipment (Thermo Fisher Scientific, Vantaa, Finland).

### Variable definitions

We defined CKD as the presence of eGFR <60 mL/min and/or albuminuria >30 mg/day in 24 h. We used the KDIGO recommended classification to determine CKD stages [16].

We defined a DM2 patient as those who previously had this diagnosis, those who were taking antidiabetic medication, those with glycosylated hemoglobin (HbA1c) >7%, those with fasting serum glucose >126 mg/dL or random serum glucose >200 mg/dL. We defined an HTN patient as the one who previously had this diagnosis, those with a systolic blood pressure > 140 mmHg or diastolic blood pressure > 90 mmHg, and those who were taking antihypertensive medication. We combined HTN and DM2 in the following classification: patients without DM2 or HTN, patients with DM2 without HTN, patients with HTN without DM2, and patients with DM2 and HTN.

We use the Body Mass Index (BMI) to define whether the patient had overweight or obesity. We classified them as normal (<25 Kg/m^2^); overweight (25Kg/m^2^ - <30Kg/m^2^), and obesity (≥30Kg/m^2^).

We also considered gender, age (in years), and serum uric acid, categorized as: <5 mg/dL, 5 to <7 mg/dL and ≥7 mg/dL.

### Statistical analysis

We used STATA v13.0 for statistical analyses. The description of the variables was performed using means, standard deviations, and absolute and relative frequencies.

We used the CKD diagnosis and its stages according to the eGFR with the MDRD-4 formula. Prevalences of CKD and albuminuria were calculated with their confidence intervals at 95% (95%CI).

To analyze the bivariate association of CKD with covariates we used chi-square test for categorical variables. Poisson regression models with robust variance were used to calculate the crude and adjusted prevalence ratios (PR) and their 95% confidence intervals (95% CI). For adjusted analysis, we only included variables that had a *p* < 0.20 in the crude analysis. We considered significant variables those with *P* values <0.05 in adjusted analyses.

## Results

**Fig. 1:**
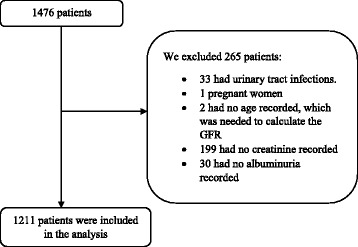
Flowchart for inclusion of study subjects. GFR: Glomerular Filtration Rate

From a total of 1476 patients, 265 were excluded for several reasons, leaving 1211 for analysis (Fig. 1). In this population, 716 (59.0%) were females and the average age was 65.8 years old (SD 12.7). There were 660 (54.5%) patients with HTN, 105 (8.7%) patients with DM2, 241 (19.9%) patients who had both diseases, and 205 (16.9%) who had neither DM2 nor HTN (Table 1).

**Table 1:** Characteristic of the population with and without CKD

Characteristics	Total	No CKD *N* = 992	CKD *N* = 219	*p*
Gender				0.004
Female	714 (59.0)	604 (84.6)	110 (15.4)	
Male	497 (41.0)	388 (78.1)	109 (21.9)	
Age (years)^a^	65.8 ± 12.7	65.1 ± 12.7	69.0 ± 11.9	<0.001
DM2 and HTN				<0.001
None	205 (16.9)	186 (90.7)	19 (9.3)	
HTN	660 (54.5)	544 (82.4)	116 (17.6)	
DM2	105 (8.7)	87 (82.9)	18 (17.1)	
HTN + DM2	241 (19.9)	175 (72.6)	66 (27.4)	
BMI				0.382
Normal	249 (20.6)	207 (83.1)	42 (16.9)	
Overweight	557 (46.0)	462 (82.9)	95 (17.1)	
Obesity	405 (33.4)	323 (79.8)	82 (20.2)	
Uric acid				<0.001
< 5 mg/dL	336 (64.7)	299 (89.0)	37 (11.0)	
5 a < 7 mg/dL	151 (29.1)	117 (77.5)	34 (22.5)	
7 a más mg/dL	32 (6.2)	16 (50.0)	16 (50.0)	

Percentages for the total column were calculated dividing for the entire population for each variable (complete 100% for each column). Percentages for the “No CKD” and “CKD” columns were calculated for each category (complete 100% for each row)

*CKD* Chronic Kidney Disease

*HTN* Hypertension

*DM2* Type 2 Diabetes Mellitus

*BMI* Body mass index

^a^Mean ± Standard deviation

Of the evaluated patients, 219 (18.1%) met the CKD criteria. Most of these patients (98.5%) correspond to the stages 1 to 3. Patients in stage 5 were 0.2% of the studied population. (Table 2) The cumulative percentages for these values are showed in the Additional file 1: Table S1.

**Table 2:** CKD stages stratified by albuminuria levels

CKD stage	Total^a^	Albuminuria^b^		
		<30 mg/g	30-300 mg/g	>300 mg/g
		*N* = 1091 (90.1%)	*N* = 105 (8.7%)	*N* = 15 (1.2%)
Stage G1	502 (41.5)	458 (91.2)	43 (8.6)	1 (0.2)
Stage G 2	581 (48.0)	534 (91.9)	40 (6.9)	7 (1.2)
Stage G3A	91 (7.5)	76 (83.5)	13 (14.3)	2 (2.2)
Stage G3B	23 (1.9)	18 (78.3)	4 (17.4)	1 (4.3)
Stage G4	11 (0.9)	3 (27.3)	5 (45.5)	3 (27.3)
Stage G5	3 (0.2)	2 (66.7)	0 (0.0)	1 (33.3)

*CKD* Chronic Kidney Disease

^a^Percentages calculted in colums

^b^Percentages calculated in rows

The prevalence of CKD was 9.3% (95% CI 5.2-13.3) in patients without HTN or DM2, 20.2% (17.6-22.8) in patients with HTN, and 24.3% (19.8 – 28.8) in patients with DM2. Also, the prevalence of albuminuria ≥30 mg/day was 3.4% (0.9-5.9) in patients without HTN or DM2, 10.8% (8.8 - 12.8) in patients with HTN, and 17.9% (13.9 - 21.9) in patients with DM2. Male patients with DM2 or HTN had higher prevalence of CKD and albuminuria (Table 3).

**Table 3:** CKD and albumuria prevalences by gender

Diagnosis	Total		Female		Male	
	Prevalence	95% CI	Prevalence	95% CI	Prevalence	95% CI
CKD						
None	9.3	5.3-13.3	9.4	4.3-14.5	9.0	2.6-15.4
HTN	20.2	17.6-22.8	16.8	13.6-20.0	25.3	20.8-29.8
DM2	24.3	19.8-28.8	22.0	16.0-28.0	26.8	20.0-33.6
Albuminuria ≥30 mg/g						
None	3.4	0.9-5.9	2.4	0.0-5.1	5.1	0.2-10.0
HTN	10.8	8.8-12.8	7.1	4.9-9.3	16.2	12.4-20.0
DM2	17.9	13.9-21.9	14.3	9.2-19.4	22.0	15.6-28.4

Patients with comorbid HTN and DM2 were included in both the HTN and the DM2 groups

*CKD* Chronic Kidney Disease

*HTN* Hypertension

*DM2* Type 2 Diabetes Mellitus

In adjusted analysis, the CKD prevalence was 2.5% higher for each year older that was the patient. It was 337% higher in DM2 patients, and 390% higher in patients with HTN and DM2 compared to patients without both diseases. Likewise, it was 104% greater in patients with serum uric acid from 5 to <7 mg/dL, and 419% greater in patients with serum uric acid ≥7 mg/dL, compared with those with <5 mg/dL (Table 4).

**Table 4:** Factors associated with CKD

Charasteristic	Crude	Adjusted^a^
	PR (95% CI)	PR (95% CI)
Gender		
Female	Ref	Ref
Male	1.42 (1.12-1.81)	0.92 (0.62-1.38)
Age (years)	1.02 (1.01-1.03)	1.03 (1.01-1.04)
Diabetes or hypertension		
None	Ref	Ref
HTN but not DM2	1.90 (1.20-3.00)	2.30 (0.91-5.80)
DM2 but not HTN	1.85 (1.01-3.37)	3.37 (1.09-10.39)
HTN + DM2	2.95 (1.84-4.75)	3.90 (1.54-9.88)
BMI		
Normal	Ref	
Overweight	1.01 (0.73-1.41)	
Obesity	1.20 (0.86-1.68)	
Uric acid		
< 5 mg/dL	Ref	Ref
5 a < 7 mg/dL	2.04 (1.34-3.13)	2.04 (1.31-3.19)
≥ 7 mg/dL	4.54 (2.86-7.20)	5.19 (3.32-8.11)

*CKD* Chronic Kidney Disease

*HTN* Hypertension

*DM2* Type 2 Diabetes Mellitus

*BMI* Body mass index

^a^Adjusted for all the variables shown, which showed a *p* < 0.20 in crude model

## Discussion

In our sample from a primary care setting, we found a global CKD prevalence of 18%. Stages 1 and 2 were the most common, and CKD was associated with age older than 73 years old, HTN, DM2, and hyperuricemia.

### CKD prevalence

In our study, 18% of the population had CKD, similar to that found by *Francis* et al. who found a CKD prevalence of 20.7% in Lima [10]. This similarity occurs despite the fact that in our study, the proportion of DM2 and older patients was higher, as we are likely to have overestimated the prevalence of CKD when using the MDRD-4 formula. It is possible that these prevalence similarities are due to the definition of albuminuria in both studies. While in our study, albuminuria was defined as >30 mg/day, Francis et al. defined it as >150 mg/g, which could increase the proportion of patients meeting the definition of CKD in our study [10]. Despite this, the prevalence of CKD in Peru is higher than other chronic diseases such as DM2, which in our country has a prevalence of 7% [17].

In a systematic review of studies that used both the KDIGO and the National Kidney Foundation: Kidney Disease Outcomes Quality Initiative (NKF KDOQI) CKD definitions, authors found an overall prevalence in people older than 20 years old of 11.8% in females and 10.4% in males. The age-standardized prevalence in low and middle-income countries was 8.6% in men and 9.6% in women [5]. Recently, another systematic review, which used the definition and classification by KDIGO, found a worldwide global CKD prevalence of 13.4% [18].

Among low and middle-income countries, a primary care center study in Sudan with a prevalence of 10% of HTN and 5.9% of DM2 found that 10.3% had a GFR < 60 ml/min and 24.1% had proteinuria [19]. In a Chilean primary care center study, CKD prevalence was 12.1% although it was not specified the frequency of HTN and DM2 patients [20].

Even though our study shows a CKD prevalence above the worldwide average and above the reported in population studies from developed countries, our prevalence is as high as the many population studies conducted in low and middle-income countries [5, 6, 21]. Mexico reported a prevalence up to 33% [7], El Salvador 18% [8], Pakistan 29,9% [22], Bangladesh 26% [23]. However, we must emphasize that the prevalence of traditional factors was variable. For example, in Jalisco the DM2 prevalence was greater than in Mexico City (44% vs. 28%) [7], while in El Salvador the DM2 prevalence was 18.5 and 30.5% in HTN [8, 24]. In the same way, the variability of the prevalence of the traditional CKD factors in primary care studies described above makes it difficult to compare with other results.

### CKD stages

In our study, the majority of patients corresponded to stages G1 to G3, as in a recent meta-analysis describing the global prevalence of CKD according the disease stages [18]. Thus, the prevalence of CKD G1 (GFR > 90 + albuminuria > 30) was 3.5%; CKD G2 (GFR 60-89 + albuminuria > 30) 3.9%; CKD G3 (GFR 30-59) 7.6%; CKD G4 (GFR 15-29) 0.4%, and CKD G5 (GFR < 15) 0.1% [18]. However, in our study there is a predominance of stages G1 and G2, unlike the G3 stage in this meta-analysis. It is possible that being primary care patients, with fewer comorbidities that merit the attention of a specialist, is the reason that the disease has been detected in the early stages.

Due to lack of reporting problems, the authors of the meta-analysis failed to divide stage 3 into the subdivision suggested by KDIGO [18]; however, in our population the largest number of patients in this stage correspond to ERC G3a (GFR 45-59), similarly to that found in other studies [16, 21].

Recent research questions the prognostic value of stage 3 in relation to progression of CKD. In the Renal Risk in Derby study, although the overall mortality of patients with CKD 3b and 4 was greater than the general population, at 5 years of follow-up, most patients with stage 3 had a stable renal function or improvement of it. These results suggest that in many patients a moderate decrease in their renal function has no implication in their renal prognosis [25].

This sub-classification has public health implications, and it reinforces the idea that more patients are found in the silent early stages of the disease. Appropriate medical intervention in these early stages decreases the risk of progression to late stages where the patient is likely to require some form of renal replacement therapy such hemodialysis (HD) [16]. This provides an opportunity to our health system, where CKD screening in at-risk population is deficient [26] and to our Ministry of Health since this institution has national HD coverage problems and has reported high mortality HD incident population, associated with late CKD detection in the Peruvian population [15, 27].

### Associated factors to CKD

The fact that both DM and HTN are related to CKD in our study is not surprising. Hill NR found that CKD is associated with DM and HTN around the world [18], and Francis et al. found similar results in a Peruvian population, and additionally found a two-fold increase in the probability for CKD in female patients with DM and HTN [10].

Among the studies with primary care patients, Temimovic in Serbia also found a higher frequency of CKD in patients with HTN and DM than in those who did not suffer from these conditions [28]. Salvador-Gonzales B in Spain found, as well, an association between HTN (OR = 2,18; 95% CI 2,08-2,30) and DM (OR = 1,26; 95% CI 1,17-1,34) and CKD [29].

The proportion of patients with CKD in the DM group and the DM/HTN group was lower that the one reported in other studies [30], and this may be related to the fact that the mean age of our population was 65,8 years, or because our patients were referred with an early onset diagnosis. These results support the need for interventions that control both DM and HTN [31].

Our study finds other factors associated with CKD such as age, obesity and hyperuricemia, in that sense the prevalence of CKD in our study was greater in those with higher age, as previously reported in Peru [10], and worldwide [5, 18]. Even though the prevalence of other risk factors for CKD (like DM and HTN) also increases with age, this association is independent [18]. It has been proposed that aging can cause a decrease in renal function, but there is not enough evidence to change the current definition of CKD according to the patient’s age [16].

Likewise, the association between overweight/obesity and CKD has biological plausibility, because of the glomerular hypertrophy that accompanies obesity and can accelerate kidney injury; nevertheless studies on the matter have discrepant results [32], as in our study. Hill NR et al. found no association between BMI or obesity and CKD [18]. The discrepancies may be related to the way that obesity is evaluated, however, regardless the relationship between obesity and CKD, there is an association with DM2 and HTN, In our country, the PERU MIGRANT study found obesity (measured by BMI) and central obesity (measured by waist circumference) prevalences of 20 and 52.5%, respectively [33].

With respect to hyperuricemia, a recent meta-analysis found a relative risk for CKD of 1.22 (95%CI 1.16–1.28, I^2^  =  65.9%) per every 1 mg/dL increased of uric acid [34]. This association is found in patients older than 60 years old. This meta-analysis excluded studies with patients with GFR less than 60 mL/min avoiding bias that hyperuricemia is due to reduce uric acid excretion by the CKD or coexisting diuretics [34]. Also, hyperuricemia is associated with other CKD risk factors such as HTN [35] and DM [36].

### Limitations

Our study has some limitations. First, we used the dataset of a referral facility in charge of providing centralized specialized evaluations for patients with hypertension and diabetes. In that sense, its coverage is broad, with an emphasis on those with a diagnosis of hypertension and/or diabetes at the primary care level of the EsSalud provider. Yet, this may introduce selection bias as not all primary-care patients are recommended to seek care at CEDHI, not all of those who are recommended do complete their annual evaluations, and, importantly, other non-EsSalud providers are not considered in this service. In general, EsSalud does provide care to approximately 30% of the peruvian population and is the one with an established care path for hypertension and diabetes. Hence, these results, are quite robust and informative and serve as an approximation to CKD in the population with HTN and DM2 being served at the primary care level. Both diseases are high-risk groups for CKD of high prevalence in our population. The HTN prevalence in Peru adjusted for age and gender is 0.65 and 0,41 to DM2 [37].

Second, we did not evaluate other known CKD associated risks such as smoking, low birth weight, etc., as well as other non-traditional risk factors found in other low and middle-income countries.

Third, to calculate the eGFR we use the MDRD-4 formula, although KDIGO suggest the CKD-EPI [16]. This was due to the fact that a non-standardized [38] method was used for the measurement of creatinine, which could overestimate the prevalence of CKD. However, in these cases is recommended the MDRD-4 formula [38], and is the formula used in 46% of studies reporting a worldwide prevalence of CKD [39]. Also, in a recent systematic review comparing the CKD prevalence with the calculated GFR using standardized vs. non-standardized creatinine measurements, no differences were found regarding worldwide prevalence [18].

Fourth, the data of our analysis were collected in 2011, and it is possible that the improvement of GDP in our country (€ 4073 in 2011 to € 5567 in 2015) should be taken into account in the current interpretation of our results.

Finally, eGFR calculation and albuminuria measurement were made once. Considering the fact that for the definition of CKD, according to KDIGO, the time between the decrease in GFR and the presence of a renal damage marker should be more than 3 months [16], our findings could overestimate the prevalence of CKD. A previous study found up to 30% of false positive rates of CKD [40].

## Conclusions

The CKD prevalence in primary care in Peru is high, and, it is more frequent in the early stages of the disease. Older patients, patients with DM2 and HTN patients, and with hyperuricemia had higher prevalence of CKD. Considering that many of the factors associated with CKD are potentially controllable factors, health authorities should design national disease control programs in order to reduce the likelihood of complications from CKD.

## Additional file

Additional file 1: Table S1. CKD stages and albuminuria levels, with cumulative percentages. (DOCX 11 kb)

## Abbreviations

BMI: Body mass index
CKD: Chronic Kidney Disease
DM2: Type 2 Diabetes Mellitus
GFR: Glomerular Filtration Rate
HTN: Hypertension
UTI: urinary tract infection

## References

[1] Coresh J, Astor BC, Greene T, Eknoyan G, Levey AS (2003). Prevalence of chronic kidney disease and decreased kidney function in the adult US population: third National Health and nutrition examination survey. Am J Kidney Dis.

[2] Hallan SI, Coresh J, Astor BC (2006). International comparison of the relationship of chronic kidney disease prevalence and ESRD risk. J Am Soc Nephrol.

[3] Hillege HL, Janssen WM, Bak AA (2001). Microalbuminuria is common, also in a nondiabetic, nonhypertensive population, and an independent indicator of cardiovascular risk factors and cardiovascular morbidity. J Intern Med.

[4] Chadban SJ, Briganti EM, Kerr PG, Dunstan DW, Welborn TA (2003). Prevalence of kidney damage in Australian adults: the AusDiab kidney study. J Am Soc Nephrol.

[5] Mills KT, Xu Y, Zhang W (2015). A systematic analysis of worldwide population-based data on the global burden of chronic kidney disease in 2010. Kidney Int.

[6] Stanifern JW, Muiru A, Tazeen H (2016). Jafar and Uptal D. Pate chronic kidney disease in low- and middle-income countries. Nephrol Dial Transplant.

[7] Obrador GT, Garcia-Garcia G, Villa AR (2010). Prevalence of chronic kidney disease in the kidney early evaluation program (KEEP) Mexico and comparison with KEEP US. Kidney Int Suppl.

[8] Orantes CM, Herrera R, Almaguer M, Brizuela EG, Núñez L (2014). Epidemiology of chronic kidney disease in adults of Salvadoran agricultural communities. MEDICC Rev.

[9] O’Donnell JK, Tobey M, Weiner DE (2011). Prevalence of and risk factors for chronic kidney disease in rural Nicaragua. Nephrol Dial Transplant.

[10] Francis ER, Kuo CC, Bernabe-Ortiz A, Nessel L, Gilman RH (2015). Burden of chronic kidney disease in resource-limited settings from Peru: a population-based study. BMC Nephrol.

[11] Stenvinkel P (2010). Chronic kidney disease: a public health priority and harbinger of premature cardiovascular disease. J Intern Med.

[12] Honeycutt AA, Segel JE, Zhuo X (2013). Medical costs of CKD in the Medicare population. J Am Soc Nephrol.

[13] Liyanage T, Ninomiya T, Jha V, Neal B, Patrice HM (2015). Worldwide access to treatment for end-stage kidney disease: a systematic review. Lancet.

[14] Sánchez-Moreno F (2014). The national health system in Peru. Rev Peru Med Exp Salud Pública.

[15] Herrera-Añazco P, Benites-Zapata VA, León-Yurivilca I, Huarcaya-Cotaquispe R, Silveira-Chau M (2015). Chronic kidney disease in Peru: a challenge for a country with an emerging economy. J Bras Nefrol..

[16] Inker LA, Astor BC, Fox CH, Isakova T, Lash JP (2014). KDOQI US commentary on the 2012 KDIGO clinical practice guideline for the evaluation and management of CKD. Am J Kidney Dis.

[17] Villena JE (2015). Diabetes Mellitus in Peru. Ann Glob Health.

[18] Hill NR, Fatoba ST, Oke JL, Hirst JA, O'Callaghan CA (2016). Global prevalence of chronic kidney disease – a systematic review and meta-analysis. PLoS One.

[19] Elsharif ME, Abdullha SM, Abdalla SM, Allaelsharif EG (2013). The magnitude of chronic kidney diseases among primary health care attendees in Gezira state, Sudan. Saudi J Kidney Dis Transpl.

[20] Zúñiga SMC, Müller OH, Flores OM (2011). Prevalence of chronic kidney disease in subjects consulting in urban primary care clinics. Rev Med Chil.

[21] De Nicola L, Zoccali C (2016). Chronic kidney disease prevalence in the general population: heterogeneity and concerns. Nephrol Dial Transplant.

[22] Jafar TH, Schmid CH, Levey AS (2005). Serum creatinine as marker of kidney function in south Asians: a study of reduced GFR in adults in Pakistan. J Am Soc Nephrol.

[23] Anand S, Khanam MA, Saquib J (2014). High prevalence of chronic kidney disease in a community survey of urban Bangladeshis: a cross-sectional study. Glob Health.

[24] Silva LC, Ordúñez P (2014). Chronic kidney disease in central American agricultural communities: challenges for epidemiology and public health. MEDICC Rev.

[25] Shardlow A, McIntyre NJ, Fluck RJ (2016). Chronic kidney disease in primary care: outcomes after five years in a prospective cohort study. PLoS Med.

[26] Tomonaga Y, Risch L, Szucs TD, Ambühl PM (2013). The prevalence of chronic kidney disease in a primary care setting: a Swiss cross-sectional study. PLoS One.

[27] Herrera-Añazco P, Benites-Zapata V, Hernandez AV, Mezones-Holguin E, Silveira-Chau M (2015). Mortality in patients with chronic kidney disease undergoing hemodialysis in a public hospital of Peru. J Bras Nefrol.

[28] Temimovic R, Rasic S, Muslimovic A (2015). High prevalence of early chronic kidney disease in high risk outpatients. Mater Sociomed.

[29] Salvador González B, Rodríguez Pascual M, Ruipérez Guijarro L, Ferré González A, Cunillera Puertolas O (2015). Enfermedad renal crónica en Atención Primaria: prevalencia y factores de riesgo asociados. Aten Primaria.

[30] Bernabe-Ortiz A, Sanchez JF, Carrillo-Larco RM, Gilman RH, Poterico JA (2017). Rural-to-urban migration and risk of hypertension: longitudinal results of the PERU MIGRANT study. J Hum Hypertens.

[31] Herrera-Añazco P (2015). V Hernandez a. Mezones Holguin E. Diabetes mellitus and diabetic nephropathy in peru. Nefrología, Diálisis y Trasplante.

[32] Chang A, Kramer H (2012). CKD progression: a risky business. Nephrol Dial Transplant.

[33] Carrillo-Larco RM, Bernabé-Ortiz A, Pillay TD, Gilman RH, Sanchez JF (2016). Obesity risk in rural, urban and rural-to-urban migrants: prospective results of the PERU MIGRANT study. Int J Obes.

[34] Zhu P, Liu Y, Han L, Xu G, Ran JM (2014). Serum uric acid is associated with incident chronic kidney disease in middle-aged populations: a meta-analysis of 15 cohort studies. PLoS One.

[35] Feig DI, Kang DH, Johnson RJ (2008). Uric acid and cardiovascular risk. N Engl J Med.

[36] Kodama S, Saito K, Yachi Y, Asumi M, Sugawara A (2009). Association between serum uric acid and development of type 2 diabetes. Diabetes Care.

[37] Miranda JJ, Herrera VM, Chirinos JA, Gómez LF, Perel P, Pichardo R (2013). Major cardiovascular risk factors in Latin America: a comparison with the United States. The Latin American consortium of studies in obesity (LASO). PLoS One.

[38] Pérez-Loredo J, Lavorato CA (2015). A Negri AL, measured and estimated glomerular filtration rate. Numerous methods of measurements (PART 1). Nefrología, Diálisis y Trasplante.

[39] Brück K, Jager KJ, Dounousi E, Kainz A, Nitsch D (2015). Methodology used in studies reporting chronic kidney disease prevalence: a systematic literature review. Nephrol Dial Transplant.

[40] Glassock RJ, Warnock DG, Delanaye P (2017). The global burden of chronic kidney disease: estimates, variability and pitfalls. Nat Rev Nephrol.

