# Supplementary material for: Prevalence of chronic kidney disease in Peruvian primary care setting
Source: BMC Nephrol. 2017 Jul 19;18:246. doi: 10.1186/s12882-017-0655-x (PMC5517816; doi:10.1186/s12882-017-0655-x)
Supplement: Additional file 1: Table S1. — CKD stages and albuminuria levels, with cumulative percentages. (DOCX 11 kb) [file 12882_2017_655_MOESM1_ESM.docx]

**Supplementary table: CKD stages and albuminuria levels, with cumulative percentages**

| **CKD Stage** | **Albuminuria** | | |
| --- | --- | --- | --- |
|  | **<30mg/g** | **30-300mg/g** | **>300mg/g** |
| Stage 1 | 458 (37.8) | 43 (3.6) | 1 (0.1) |
| Stage 2 | 534 (44.1) | 40 (3.3) | 7 (0.6) |
| Stage 3A | 76 (6.3) | 13 (1.1) | 2 (0.2) |
| Stage 3B | 18 (1.5) | 4 (0.3) | 1 (0.1) |
| Stage 4 | 3 (0.2) | 5 (0.4) | 3 (0.2) |
| Stage 5 | 2 (0.2) | 0 (0.0) | 1 (0.1) |

Percentages were calculated over the whole included population
